# The Vaping and Patterns of e-Cigarette Use Research Study: Protocol for a Web-Based Cohort Study

**DOI:** 10.2196/38732

**Published:** 2023-03-02

**Authors:** Jeffrey J Hardesty, Elizabeth Crespi, Qinghua Nian, Joshua K Sinamo, Alison B Breland, Thomas Eissenberg, Kevin Welding, Ryan David Kennedy, Joanna E Cohen

**Affiliations:** 1 Institute for Global Tobacco Control Department of Health, Behavior and Society Johns Hopkins Bloomberg School of Public Health Baltimore, MD United States; 2 Center for the Study of Tobacco Products Department of Psychology Virginia Commonwealth University Richmond, VA United States

**Keywords:** internet, web-based, cohort, survey, e-cigarettes, electronic nicotine delivery systems, ENDS, tobacco, recruitment, data collection, strategies, lessons learned, mobile phone

## Abstract

**Background:**

In total, 3.2% of American adults report using e-cigarettes every day or some days. The Vaping and Patterns of E-cigarette Use Research (VAPER) Study is a web-based longitudinal survey designed to observe patterns in device and liquid use that suggest the benefits and unintended consequences of potential e-cigarette regulations. The heterogeneity of the e-cigarette devices and liquids on the market, the customizability of the devices and liquids, and the lack of standardized reporting requirements result in unique measurement challenges. Furthermore, bots and survey takers who submit falsified responses are threats to data integrity that require mitigation strategies.

**Objective:**

This paper aims to describe the protocols for 3 waves of the VAPER Study and discuss recruitment and data processing experiences and lessons learned, including the benefits and limitations of bot- and fraudulent survey taker–related strategies.

**Methods:**

American adults (aged ≥21 years) who use e-cigarettes ≥5 days per week are recruited from up to 404 Craigslist catchment areas covering all 50 states. The questionnaire measures and skip logic are designed to accommodate marketplace heterogeneity and user customization (eg, different skip logic pathways for different device types and customizations). To reduce reliance on self-report data, we also require participants to submit a photo of their device. All data are collected using REDCap (Research Electronic Data Capture; Vanderbilt University). Incentives are US $10 Amazon gift codes delivered by mail to new participants and electronically to returning participants. Those lost to follow-up are replaced. Several strategies are applied to maximize the odds that participants who receive incentives are not bots and are likely to possess an e-cigarette (eg, required identity check and photo of a device).

**Results:**

In total, 3 waves of data were collected between 2020 and 2021 (wave 1: n=1209; wave 2: n=1218; wave 3: n=1254). Retention from waves 1 to 2 was 51.94% (628/1209), and 37.55% (454/1209) of the wave 1 sample completed all 3 waves. These data were mostly generalizable to daily e-cigarette users in the United States, and poststratification weights were generated for future analyses. Our data offer a detailed examination of users’ device features and specifications, liquid characteristics, and key behaviors, which can provide more insights into the benefits and unintended consequences of potential regulations.

**Conclusions:**

Relative to existing e-cigarette cohort studies, this study methodology has some advantages, including efficient recruitment of a lower-prevalence population and collection of detailed data relevant to tobacco regulatory science (eg, device wattage). The web-based nature of the study requires several bot- and fraudulent survey taker–related risk-mitigation strategies, which can be time-intensive. When these risks are addressed, web-based cohort studies can be successful. We will continue to explore methods for maximizing recruitment efficiency, data quality, and participant retention in subsequent waves.

**International Registered Report Identifier (IRRID):**

DERR1-10.2196/38732

## Introduction

### Background

In the United States, 3.2% of adults, 7.6% of young adults [1], and 11.3% of high school students [2] use e-cigarettes (every day or some days for adults and use in the past 30 days for high school students), and the US Food and Drug Administration Center for Tobacco Products is interested in data-driven regulations that maximize public health benefits while minimizing unintended consequences [3].

Our team is conducting a web-based longitudinal survey to observe patterns that suggest the benefits and unintended consequences of three potential e-cigarette regulations: (1) limits on nicotine, (2) constraints on nicotine flux (ie, nicotine emitted over time) [4], and (3) reduction in flavor availability. Electronic nicotine delivery systems (ENDS) are e-cigarettes that heat a nicotine liquid into an aerosol that can be inhaled by a user, whereas electronic nonnicotine delivery systems (ENNDS) heat a nonnicotine-containing liquid (ENNDS are included in the study as ENDS users may become ENNDS users and vice versa). Both include a battery that powers a heating element, such as a metal coil, which is in contact with the liquid. Device size, shape, materials, features (eg, coil modifiability), and specifications (eg, battery voltage, coil resistance, and device wattage) vary considerably. The liquids are typically made of a propylene glycol and vegetable glycerin solution that contains flavorings and nicotine, a psychoactive and addictive drug [5].

Unlike cigarettes, which are all relatively similar in design, the heterogeneity of ENDS and ENNDS devices, settings, and liquid characteristics results in a highly customizable user experience. This heterogeneity and a lack of standardized reporting requirements for device specifications and liquid characteristics can lead to measurement challenges. For example, some manufacturers report liquid nicotine concentration as a percentage of the liquid solution, whereas others report it in milligrams per milliliter. These inconsistencies may partially explain why reporting liquid nicotine concentration accurately in surveys is challenging for some users [6,7]. In addition, liquid manufacturers sometimes inaccurately label nicotine concentration [8-10], and device manufacturers often do not publicize specifications such as device wattage, voltage, and resistance, thus creating additional challenges for researchers even after collecting the brand and model of a user’s device. These data are critical for evaluating nicotine and toxicant emissions and delivery. For example, device wattage is a predictor of nicotine emissions and delivery to the blood [11,12].

Web-based survey methods also present challenges that must be addressed in both a preventive and ad hoc manner; such challenges include recruitment of lower-prevalence populations and navigating bots [13] and fraudulent survey takers [14], who primarily aim to deceive researchers for the purpose of receiving incentives. Nevertheless, web-based survey methods are an increasingly used avenue to recruit participants, collect data, and provide incentives across public health research domains, including ENDS and ENNDS research, and they may have several benefits over traditional methods, such as convenience for participants and researchers, scalability, reduced costs, and safety during extraordinary times such as the COVID-19 pandemic.

Studies that address these measurement and data integrity (eg, bots) challenges can offer a more detailed examination of frequent ENDS and ENNDS users’ device features and specifications, liquid characteristics, and user behaviors while simultaneously benefiting from improved convenience, scalability, reduced costs, and safety. Moreover, through an improved understanding of the relationships among device type, features, specifications, liquid characteristics, and key user behaviors such as nicotine dependence, regulators may gain a more precise understanding of how regulating devices and liquids may positively or negatively affect users before implementing a regulation.

### Objectives

Our aim is to describe the protocols for waves 1 to 3 of the Vaping and Patterns of E-cigarette Use Research (VAPER) Study, a web-based longitudinal cohort study of ENDS and ENNDS users (aged ≥21 years) who use devices ≥5 days per week and have a residential address in the United States. We also discuss our recruitment and data processing experiences and lessons learned, along with the benefits and limitations of implementing our strategies for mitigating measurement-, bot-, and fraudulent survey taker–related challenges.

## Methods

### Overview

The protocols for waves 1 to 3 are similar. Key differences will be addressed; where none are discussed, similar protocols were followed in all waves and will also be followed in future waves. Additional technical details can be found in Multimedia Appendix 1. All data are collected using REDCap (Research Electronic Data Capture; Vanderbilt University), a free, secure, and robust data collection platform.

**Figure 1:**
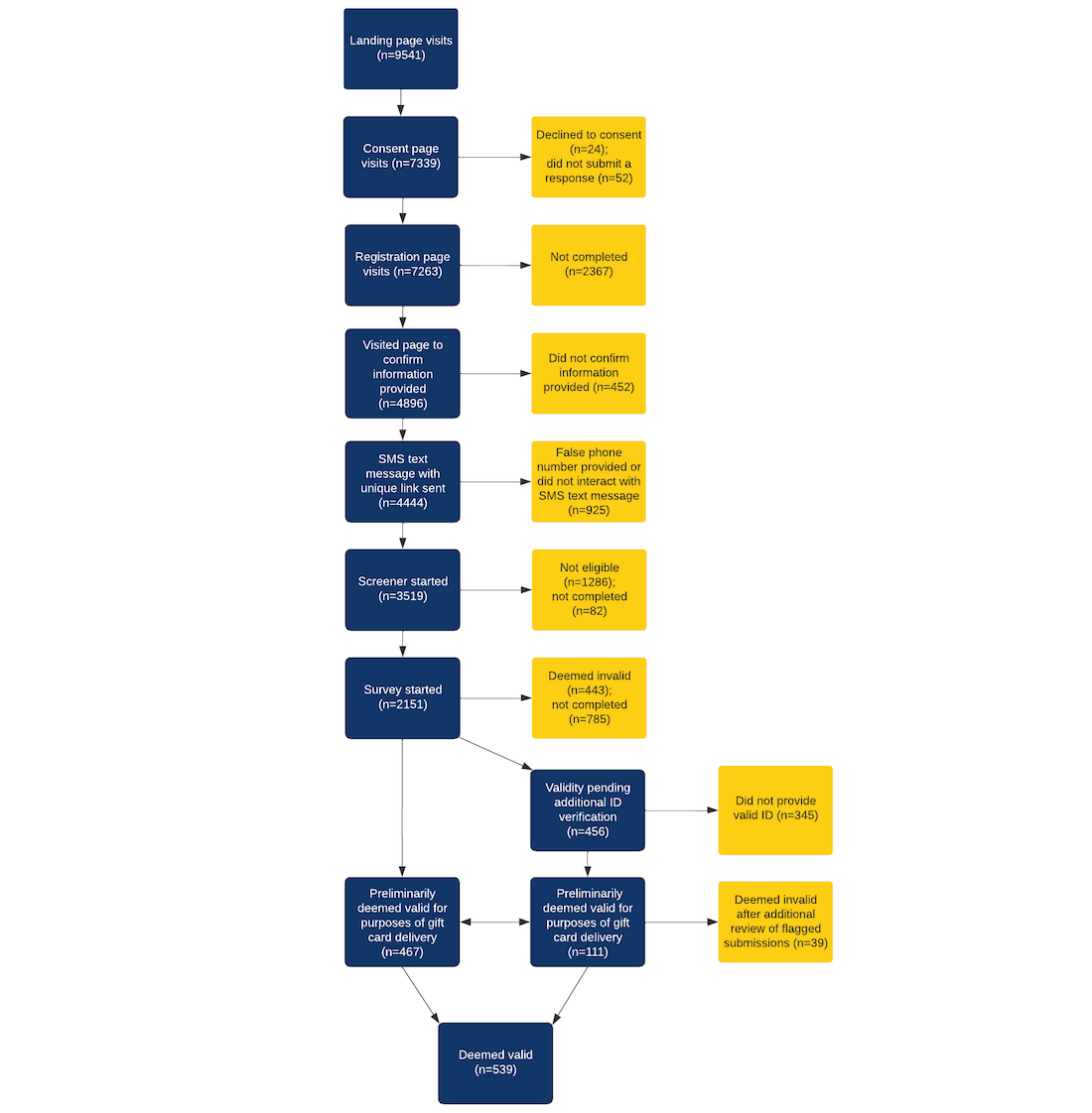
Flow diagram of baseline survey participants from wave 3.

The VAPER Study is a cohort study conducted on the web, including participant recruitment, data collection, and incentive delivery to participants. A self-selection sampling method is used. Recruitment-related information for the baseline survey is posted on Craigslist Jobs and Gigs boards and directs potential participants to click a hyperlink to a study-specific landing page with a welcome message hosted on the Virginia Commonwealth University website (the web page is not accessible through other avenues unless the hyperlink is shared by participants). After providing informed consent, participants complete a registration form requesting the following information: name, email address, mobile phone number, residential address, and date of birth. Participants then review the information they provided and complete a phone number authentication that contains a unique link to the REDCap screener and survey. Before starting the survey, all participants are reminded of actions that can result in their disqualification (also present in the consent form). Upon survey completion, identity verification, and review of submitted data, participants are mailed a US $10 Amazon gift code (Figure 1).

**Figure 2:**
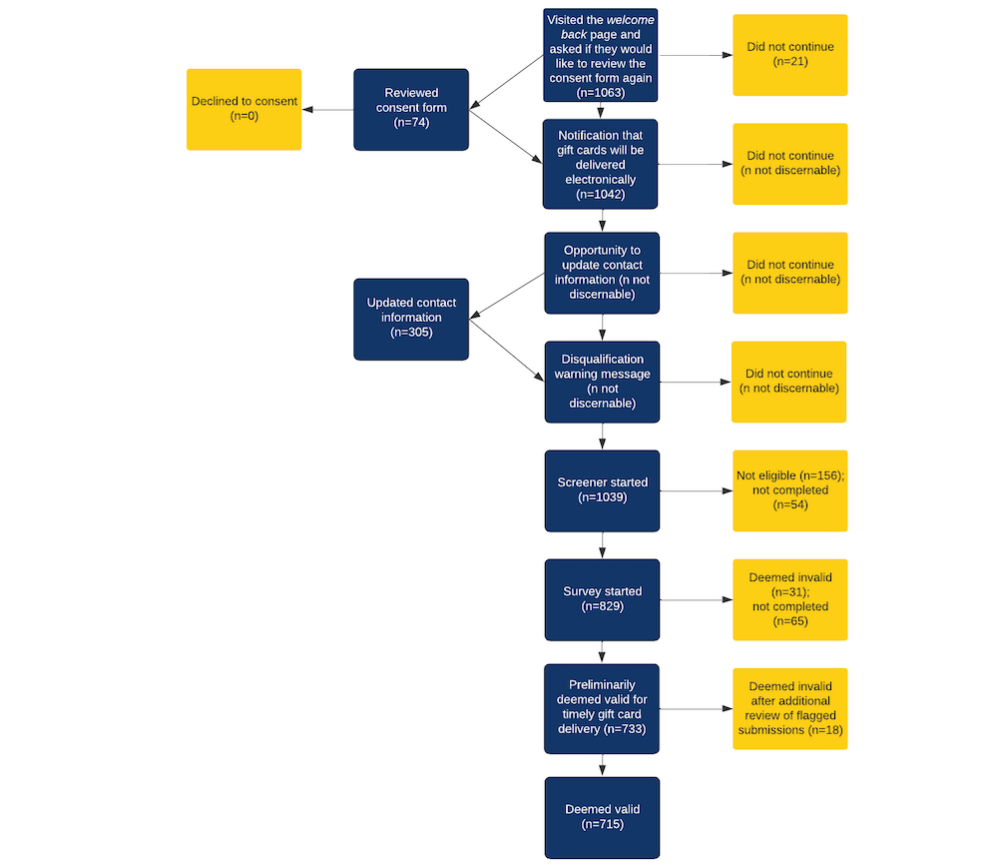
Flow diagram of follow-up survey participants from wave 3.

Participants who submit valid baseline surveys and indicate an interest in participating in additional surveys are invited to complete a follow-up survey in subsequent waves. Invitations are sent to their mobile phones and email addresses, and the links to the survey are tied to their previously established record ID number. Returning participants are greeted with a *welcome back* message and, before completing the screener, are provided with the opportunity to review the consent form again, are notified that they will receive their gift codes electronically, are asked to review and update their contact information (if necessary), and are again reminded of actions that can result in their disqualification (Figure 2).

To replace those lost to follow-up, new participants are recruited to complete the baseline survey, resulting in 2 concurrent REDCap surveys per wave: one for baseline participants and another for follow-up participants. Therefore, a new baseline cohort is created in each wave, with all previously established cohorts taking a concurrent and identical follow-up survey (Table 1). The measures in the questionnaire evolve as changes in the marketplace are recognized, as new information is learned about the quality of the questions and response options, and as the VAPER Study team fields requests from the funders and colleagues working on related laboratory-based projects. This evolution creates a layer of complexity when analyzing data longitudinally but will not affect cross-sectional analyses. For longitudinal data analyses, steps are taken to ensure that the underlying questions and response options are comparable. More specifically, a data workbook is used to track variable names for each wave and survey (ie, baseline and follow-up surveys); whether edits have been made to the question or response option text; and if “yes,” what those edits were. Manuscript authors can then determine whether their measures can be used across waves in consultation with our statistician and wider team.

**Table 1:** Surveys taken in each wave, by cohort.

	Wave 1: May 2020-October 2020	Wave 2: December 2020-April 2021	Wave 3: September 2021-December 2021
Cohort 1	Baseline	Follow-up	Follow-up
Cohort 2	N/A^a^	Baseline	Follow-up
Cohort 3	N/A	N/A	Baseline

^a^N/A: not applicable.

### Sample

Participants are ENDS and ENNDS users who typically use e-cigarettes at least 5 days per week, are aged ≥21 years, and have a residential address in the United States. Adults are recruited for several reasons: (1) more adults (10.9 million) [15] than youth (2.2 million) [16] use ENDS and ENNDS; (2) school-based recruitment methodologies are slower, more expensive, and less scalable; and (3) most of our hypothesized relationships among devices, liquids, and user behaviors are expected to be present irrespective of age (eg, a negative correlation between device power and liquid nicotine concentration). The age of 21 years was chosen over 18 years because of institutional review board (IRB) guidance and state and federal legislation that raised the minimum age of purchase to 21 years. Users who use “At least five days per week” are recruited as we are primarily interested in within-person longitudinal data. Our rationale is that frequent ENDS and ENNDS users are more likely to continue using and remain in the sample compared with users using <5 days per week, thereby increasing the quality of the longitudinal data.

The intended sample size (N=900) was determined by assuming an effect size of 10% (for *t* tests to detect differences between 2 dependent means using a 2-tailed test), Cronbach α<.05, and power of 0.85. After adjusting for an anticipated loss to follow-up rate of 25%, we determined that a baseline sample of 1200 participants was required to ensure adequate power for the study duration.

### Recruitment

**Figure 3:**
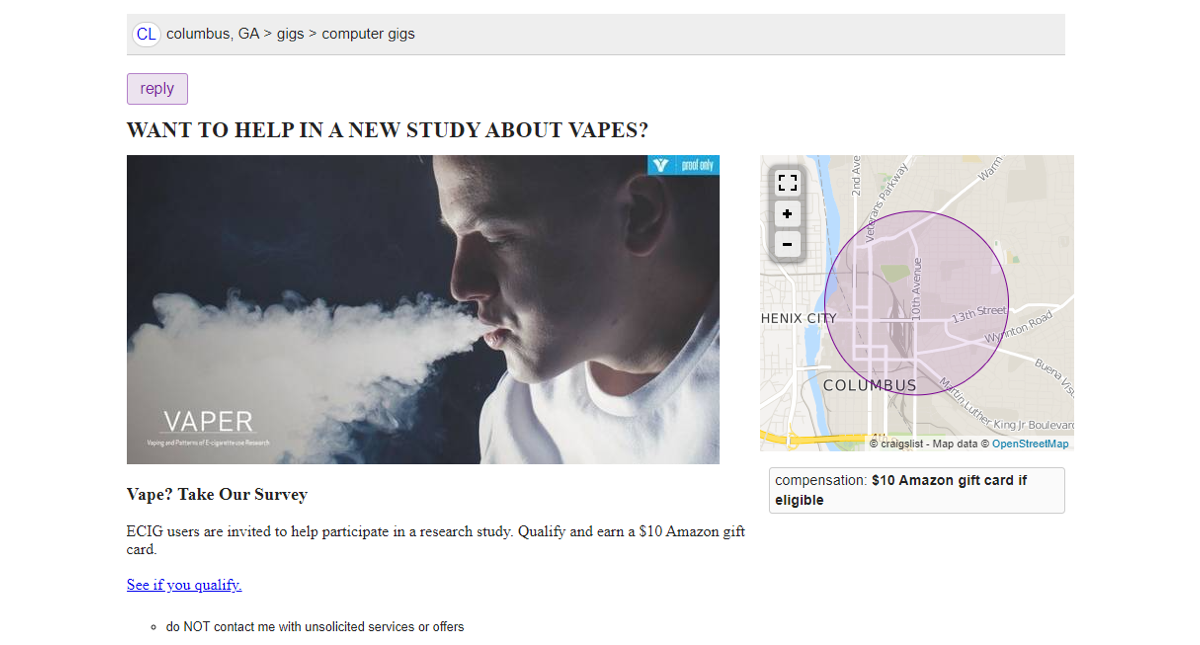
An example Craigslist posting from wave 3.

Craigslist is used to recruit baseline survey participants. It has a high volume of website traffic, a user base interested in earning income, affordable rates for posting messages targeted to potential participants in the United States, and a track record of success in helping tobacco control [17] and other public health researchers [18] recruit participants. Craigslist postings include a photo and text indicating that we are recruiting ENDS and ENNDS users and will compensate participants with a US $10 Amazon gift code (Figure 3). Note that social media (ie, Facebook and Instagram) and vape shop customer recruitment were attempted early in wave 1 but, because of cost and efficiency reasons, were replaced by a Craigslist-focused strategy. Craigslist was used to recruit most of our wave 1 sample and to replace all participants lost to follow-up in waves 2 and 3.

The Craigslist postings are posted on the Jobs and Gigs boards in as many as 404 geographic locations per wave, including all 50 US states (Multimedia Appendix 1). Both boards were used in most geographic locations, but sometimes only 1 board was used. Geographic locations were selected based on population estimates of major US cities and states, with preference given to the most populous catchment areas (note that Craigslist regions can cover a single city, regions, or large geographic areas spanning entire states). To optimize recruitment efficiency, Craigslist postings are reposted at varying time intervals primarily based on the number of competing advertisers displacing our postings to a lower position on the page (eg, New York City boards need to be reposted 2 times per week, whereas others are reposted as infrequently as once per month). Other metrics are tracked and considered as well, including the fluctuating volume of clicks from different locations over the course of a survey wave (measured in real time using Google Analytics) and the number of participants completing the survey from each location (higher-yielding locations tend to be reposted more frequently).

### Communication Strategy for Maximal Retention

To invite participants who previously submitted valid surveys to complete additional survey waves, our team sends 2 pairs of emails and SMS text messages per week for 2 weeks at the start of each wave. Afterward, 1 pair of emails and SMS text messages is sent per week for 6 weeks to all participants who have not yet completed the survey. The subject lines and message content of the reminders vary from week to week to attempt to appeal to different audiences. Note that, at the start of wave 2, tests were conducted to select the most effective communication content (eg, varied email subject lines) and frequency of communications (ie, 1 vs 2 pairs of emails and SMS text messages sent per week for 2 weeks, with each pair sent simultaneously). The tests were conducted using a small subset of the wave 1 sample (n=148), and we found no statistically significant differences in valid survey completion rates or the rates of opting out of SMS text messages, and the absolute number of SMS text message opt-outs was minimal (n=5).

### Engagement Strategy

Loss to follow-up is common in longitudinal studies. To increase engagement and minimize loss to follow-up, our participants are sent an annual postcard indicating that they are eligible for a raffle. The annual raffle has 4 winners, with each receiving a US $100 Amazon gift code. Completing 2 surveys will earn participants 10 chances to win the raffle, and completing 3 surveys will earn them 50 chances.

### Measures

Measures in the questionnaire are from or derived from those found in the PhenX Toolkit (RTI International; a web-based catalog of high-priority measures), validated measures, or measures used in large national surveys whenever possible and appropriate. We developed measures in all other instances. Randomization of questions and response options is not used; however, adaptive questioning to reduce the number and complexity of the questions is used (eg, questions are tailored based on self-reported device features). The number of questions per page is limited to 1 whenever possible; however, it is necessary to have more than 1 question per page when participants are asked to further specify information or in instances where the questions are easier to respond to in sequence (eg, grid-style questions). The number of pages varies widely based on the devices and liquids used and related behaviors. To ensure completeness, all participants must provide a response to continue to the next page (an error message is received when participants attempt to continue without completing all the questions). To mitigate data integrity issues, a back button is not used.

The usability and technical functionality of the questionnaire are rigorously tested by the study team before fielding the questionnaire in each wave. All permutations of skip logic and response option constraints are tested, and before the first wave, mock participants not familiar with the study completed the questionnaire and provided feedback on wording of the prompts, questions, response options, functionality of specific features (eg, photo upload), and overall user experience. Although these actions greatly mitigate technical issues and improve user experience, participants who complete the study during each wave often provide comments and suggestions that are incorporated as well.

To discern the impacts of potential regulations, outcome measures include current ENDS and ENNDS use (≥5 days per week), current combustible cigarette use (past 30 days), product switching (eg, devices, device settings, and liquid nicotine concentration and flavor), do-it-yourself flavor mixing (ie, mixing their own flavored solution typically by mixing a combination of propylene glycol, vegetable glycerin, nicotine, and flavorings), ENDS dependence, and respiratory symptoms. Psychosocial mediators include quitting intentions, perceived risk and severity, and outcome expectancies related to use experience. Moderators include sociodemographic factors, tobacco cigarette history, ENDS and ENNDS history, and reasons for use. Full details for select measures are available in the following sections; the full questionnaire is available on our study website [19].

ENDS and ENNDS use was assessed using the following question: “How many days in a typical week do you use an e-cigarette or vaping device to vape e-liquids with or without nicotine?” Response options included “I do not use an e-cigarette or vaping device to vape e-liquids with or without nicotine in a typical week,” “1 day,” “2 days,” “3 days,” “4 days,” “5 days,” “6 days,” and “7 days.”

Dependence is measured using the E-cigarette Dependence Scale. Participants receive the following prompt: “The following questions are about your E-CIGARETTE use only. Please respond to each question or statement by marking the most appropriate response.” The statements include “I find myself reaching for my e-cigarette without thinking about it,” “I drop everything to go out and buy e-cigarettes or e-juice,” “I vape more before going into a situation where vaping is not allowed,” and “When I haven’t been able to vape for a few hours, the craving gets intolerable.” Response options include “Almost Always,” “Often,” “Sometimes,” “Rarely,” and “Never.”

Quitting intentions are assessed using the following questions: “Are you planning to quit vaping” and “Are you planning to quit smoking cigarettes.” Response options include “Within the next month,” “Between 1-6 months from now,” “Sometime in the future, beyond 6 months,” and “Not planning to quit.” For cigarettes, only those who indicate that they have smoked ≥100 cigarettes in their lifetime and a cigarette in the past 30 days are asked the question.

The questions and skip logic are designed to accommodate marketplace heterogeneity and user customization. For example, there are different skip logic pathways for participants with disposable devices; disposable pod– or cartridge-based devices; and refillable pod–, cartridge-, or tank-based devices. These pathways allow our team to tailor the questionnaire to each participant’s situation and experiences with the expectation of creating a better user experience for the participants, higher retention, and higher-quality data. Another byproduct of this approach is that it allows for inquiry about the devices, liquids, and related behaviors that do not apply to all participants, such as the addition of extra nicotine to one’s liquid (often called “nicotine boosters” or “nicotine shots”). Such behavior would only be applicable to participants who refill their device from a bottle of liquid. Please note that these device and liquid questions are not validated.

### Device and Liquid Data Collection

Previous studies have suggested that self-report data alone may not be a viable strategy for capturing accurate device and liquid data [6,7]. To minimize reliance on these data, we require participants to submit valid photos of their most commonly used device, the current visual display screen (powered on) if available, and the most commonly used liquid for the device if available. Following a standard operating procedure, submitted photos are reviewed to identify the brand and model of the device. When the brand or model are not immediately apparent or found with the aid of a Google search, we use unique features, colors, and text on the device to conduct a Google image search. Upon identifying the brand and model, key variables are collected from manufacturer, academic, retailer, and review websites. YouTube product reviews are also helpful in understanding whether certain features are present when information is not readily available on the aforementioned websites (eg, adjustable airflow). To mitigate issues related to inconsistent reporting of device features and specifications and liquid characteristics across websites, data are collected preferentially from (1) manufacturer sites, (2) academic manuscripts, (3) retailer sites, (4) review sites, and (5) YouTube. Key variables collected for devices include wattage, voltage, resistance, coil modifiability, power modifiability, airflow adjustability, disposable versus reusable, and pod- or cartridge-based device versus tank-based device. Liquid variables include brand, flavor (primary and secondary), container size (mL), nicotine concentration (mg/mL), nicotine formulation (free base vs protonated), propylene glycol percentage, and vegetable glycerin percentage.

### Missing Data

Missing data is a multifaceted issue. Participants may self-report not knowing the details of the products used and their related settings and specifications. Photos are also vulnerable to user error, with some participants not following instructions (eg, blurry images), resulting in unidentifiable device brands and models, visual display settings, and liquid characteristics. In addition, some web-based sources do not report all device features, specifications, and liquid characteristics.

We use several preventive strategies to mitigate these issues. First, by collecting device and liquid data via self-report and from photos and web-based sources and creating a combined variable that prioritizes photo and web-based data, we minimize missing data and reliance on participant expertise on the product details. Furthermore, participants are required to answer each question to advance and complete the questionnaire, resulting in minimal missing data for all questions without “Don’t know” and “Prefer not to answer” response options (responses with identifying information were removed). Finally, for the collection of data from photo and web-based sources, participants are provided with instructions for submitting photos, and a comprehensive standard operating procedure is adhered to by the team to ensure that web-based data are reliably collected.

Despite these preventive efforts, missing data can still occur; therefore, we implement 3 additional post hoc strategies specific to device wattage, voltage, and coil resistance. The first strategy is to purchase the most prevalent devices in the sample with missing data for 2 or more of these device specifications, disassemble the devices, and directly measure the voltage and resistance using a multimeter when one or both values are missing. The second strategy is to use a power calculator (ie, a mathematical formula for calculating wattage, voltage, or resistance) when a single specification is missing. The third strategy is used when three conditions are met: (1) the device’s range (eg, 0-80 W) is known, (2) the participant’s current setting within the range is unknown (eg, the picture is blurry or there is no visual display), and (3) the setting is not self-reported by the participant. Under these conditions, we estimate the participant’s setting by calculating the average midpoint for the device type (eg, the average refillable tank user used 35% of the allowable wattage range in wave 3). The average midpoint (eg, 35%) is then applied to the specific device’s known range.

### Data Integrity and Security

Rigorous measures are taken to prevent bots and fraudulent survey takers from subverting data integrity. All participants who register and complete the baseline survey provide identifying data and agree to participate in identity verification procedures. LexisNexis (RELX corporation), a third-party identity search engine, is used to verify identities before providing incentives. In instances where the LexisNexis database is not sufficient to identify participants, they are asked to provide a photo ID or utility bill that contains information confirming their identity.

Bot protections include a Completely Automated Public Turing test to tell Computers and Humans Apart (CAPTCHA), authentication of a mobile phone number, attention-checking questions, disabling the back button, and manual review of open-ended responses. The CAPTCHA is a well-known test that requests users to perform simple tasks that humans can accomplish easily but unsophisticated bots cannot. Authentication is used to confirm that phone numbers are real and to generate a new randomly generated survey link. By having 2 REDCap forms linked in this way, bots cannot easily generate new identities and immediately proceed to take the survey. Attention-checking questions not only help us identify participants quickly moving through the survey but also help identify unsophisticated bots who may be providing random answers to the questions. The back button is disabled to prevent participants who build bots from more easily learning the questionnaire and skip logic, thus making it more time-intensive to build a sophisticated bot capable of providing answers that closely mimic real users. The manual review of open-ended responses is particularly important as bots often use similar responses across multiple survey submissions. By screening the new survey submissions on a weekly basis for repeated phrases (particularly uncommon phrases), spelling, and formatting errors, we can more easily identify problematic cases and adjust the strategy as needed. However, the most sophisticated bots may be capable of circumventing these procedures and may be indistinguishable from real users. These strategies are meant to mitigate the chances of having bot-related issues.

Fraudulent survey takers are participants who are savvy enough to enroll in and complete surveys for which they are not qualified. Similar to bots, they may also attempt to take a survey multiple times. By using identity checks, the risk that participants will take the survey multiple times is lower (note that they can use another person’s identity); however, they may not be truthful about their e-cigarette use behavior. To further mitigate the risk of enrolling fraudulent survey takers, we use clear warnings against misuse, require participants to submit a photo of their most used device from the past week, review each photo to verify that it is valid (ie, instructions were followed), and mail incentives. Our warning statement is placed in the consent form and at the start of the surveys: “As a reminder, any perceived attempt to speed through the survey, take the survey more than once, or provide false or misleading information will result in your disqualification from the survey and forfeiture of any promised incentives.” This provides us with maximum flexibility for determining who should not receive an incentive and be included in our final sample. Requiring that participants submit a photo of their most used device from the past week is essential to ensuring that they are in possession of a device and, thus, more likely to be ENDS or ENNDS users. Photo submissions of their most commonly used devices from the past week are rigorously reviewed for the following evidence: the objects featured in the photo are not ENDS or ENNDS devices, the photos are downloaded from the internet, the photos are staged in a store, the photos are identical or nearly identical to submissions by other unique IDs, and the photo-coded brand and model do not match the self-reported brand and model for 3 devices that were provided as examples in the self-report brand and model question; that is, “What is the brand AND model of the device (e.g., JUUL, Vaporesso Luxe, Voopoo Drag 2, etc.)?”

Other rigorous data quality checks are also performed before participants receive their incentives. The checks include identifying the use of non-English or non-Spanish alphabet characters in open-ended responses; verifying that a proper mailing address has been provided; confirming that participants do not submit more than 1 survey within or across survey waves; and verifying that more than the minimal number of questions have been answered (skip logic is such that this is highly improbable), completion time is >5 minutes, and 2 attention-checking questions are answered appropriately (both require correct responses).

Data security is ensured through the use of REDCap and Twilio. REDCap is a secure web application that is used to build web-based surveys and databases. It collects any type of data and is geared toward supporting data capture on a server for category-1 data (ie, confidential data). Access to files with identifying information is restricted to approved research team members, all of whom are trained in standards of research privacy and confidentiality and who have secure passwords required to sign onto the data server. When off-campus, virtual private network services are used to access the server. Twilio is a web service used to send a private survey link to respondents. Participants’ phone numbers do not remain in Twilio’s logs but are removed shortly after being completed, which is done for security and privacy concerns.

### Incentives

The incentive for completing the 15-minute survey is a US $10 Amazon gift code. Once submissions are determined to be preliminarily valid after the initial data quality review and participants’ identities are confirmed, incentives are mailed to the physical address provided by baseline survey participants and emailed to follow-up survey participants. The data quality check and identity verification are typically completed within 3 days of submission, and incentives are mailed on a weekly basis. Baseline survey participants are mailed their incentives as a form of delayed gratification to deter them from attempting to take the survey multiple times. Post office box addresses and other nonresidential mailboxes are not accepted to prevent multiple submissions. Occasionally, incentives are returned to the sender, indicating that a false address was provided, the participant moved, or a typo was present in the provided address. These record IDs are reviewed more closely for other data quality issues (eg, responses indicating that they were likely not ENDS or ENNDS users). If other survey data are found to be of low quality, the corresponding participants are dropped from the data set and not invited to future waves but are still eligible to receive their incentive for the wave in question. To facilitate the emailing of gift codes to follow-up participants, Rybbon (BHN Rewards), a digital gift code delivery service, is used.

### Final Data Quality Checks

Once data cleaning procedures are completed after each survey wave, additional data quality checks are completed when preliminarily valid records are flagged. These include instances of photos with multiple devices or liquids present, poor photo quality, photos or survey responses with non–propylene glycol and vegetable glycerin solutions (eg, tetrahydrocannabinol or cannabidiol), age of first use occurring before ENDS and ENNDS were commercially available in the United States (baseline only), select examples of REDCap skip logic not working as intended (investigated by REDCap; the software bug remains unknown), and incentives being returned to the sender (as previously described). When at least one flag is identified, all the survey responses are reviewed for additional evidence of poor data quality. For example, additional evidence may include the self-reported device brand and model not matching the photo brand and model or self-reported liquid flavors not matching their respective photos. A scoring system is used to determine whether the issues found warrant exclusion from the current and future waves of data collection. All excluded participants still receive their incentive for the survey wave in question.

### Ethics Approval

The IRB at the Virginia Commonwealth University (HM20015004) approved the study protocol. The Johns Hopkins Bloomberg School of Public Health IRB (9277) approved reliance on the Virginia Commonwealth University IRB.

## Results

### Recruitment and Retention

Funding for the VAPER Study began on September 1, 2018, and will conclude on August 31, 2023. Data collection for waves 1 to 3 was completed over 3 periods. Wave 1 was completed between May 18, 2020, and October 16, 2020 (n=1209); wave 2 was completed between December 10, 2020, and April 21, 2021 (n=1218); and wave 3 was completed between September 2, 2021, and November 18, 2021 (n=1254). Partially completed questionnaires are not analyzed.

Upon conclusion of wave 2, the retention rate of cohort 1 (baseline survey participants from wave 1; n=1209) was 51.94% (628/1209), and 5.29% (64/1209) of participants opted out of SMS text message reminders. For wave 3, a total of 37.55% (454/1209) of cohort 1 completed the survey, with 33.25% (402/1209) completing the wave 2 and 3 surveys (52/1209, 4.3% completed the survey for wave 3 but not for wave 2). A total of 7.03% (85/1209) of participants opted out of SMS text message reminders. Cohort 2 (baseline survey participants from wave 2; n=590) had a wave 3 retention rate of 44.2% (261/590), and 6.4% (38/590) of participants opted out of the SMS text message reminders.

### Generalizability

For each wave, our wave 1 to 3 frequent users were largely generalizable to daily users of e-cigarettes in the United States (1185/1254, 94.5% of our wave 3 sample used ENDS or ENNDS 7 days per week; Table 2). There was no statistically significant difference between our wave 3 sample and the weighted 2019 Tobacco Use Supplement to the Current Population Survey data in terms of *age/gender/race* (*P*=.18) and *region* (*P*=.42). Compared with the Tobacco Use Supplement to the Current Population Survey, our wave 3 sample had a higher percentage of frequent ENDS or ENNDS users with an income of US <$60,000 (928/1225, 75.76% vs 804,024/1,537,547, 52.3%; *P*<.001). Applying poststratification weighting can help improve the representativeness of the data.

**Table 2:** A comparison of wave 3 and Tobacco Use Supplement to the Current Population Survey (TUS-CPS) 2019 frequencies for 3 weighting strategies.

			VAPER^a^—wave 3, n (%)		TUS-CPS 2019 (n=554)				
					Participants, n (%)		Weighted N (%)		SE of weighted frequency
**Gender, age, and race (n=1233)^b^**									
	Men, <35 years, and non-White^c^	92 (7.5)		16 (2.9)		55,388 (3.6)		15,766	
	Men, <35 years, and White	216 (17.5)		139 (25.1)		370,806 (24.2)		33,224	
	Men, ≥35 years, and non-White	48 (3.9)		14 (2.5)		46,345 (3)		14,129	
	Men, ≥35 years, and White	183 (14.8)		159 (28.7)		414,366 (27)		32,117	
	Women, <35 years, and non-White	100 (8.1)		10 (1.8)		41,041 (2.7)		13,602	
	Women, <35 years, and White	250 (20.3)		85 (15.3)		243,554 (15.9)		28,760	
	Women, ≥35 years, and non-White	55 (4.5)		11 (2)		36,008 (2.4)		12,207	
	Women, ≥35 years, and White	289 (23.4)		120 (21.7)		327,967 (21.4)		29,119	
**Annual income (US $)^d^ (n=1225)^e^**									
	<60,000	928 (75.8)		287 (51.8)		804,024 (52.3)		42,114	
	≥60,000	297 (24.2)		267 (48.2)		733,523 (47.7)		39,627	
**Region (n=1254)**									
	Northeast	172 (13.7)		89 (16.1)		258,265 (16.8)		28,287	
	Midwest	263 (21)		134 (24.2)		427,656 (27.8)		36,026	
	South	465 (37.1)		192 (34.7)		548,960 (35.7)		37,425	
	West	354 (28.2)		139 (25.1)		302,665 (19.7)		28,726	

^a^VAPER: Vaping and Patterns of E-cigarette Use Research.

^b^Missing data: n=21.

^c^White includes single race White; non-White includes all other single races, including American Indian or Alaska Native, Asian or Asian American, Black or African American, Native Hawaiian or Pacific Islander, other, and multirace.

^d^Denotes a statistically significant difference between VAPER and TUS-CPS data at *P*<.001.

^e^Missing data: n=29.

Poststratification weighting normally requires the sample size of a subgroup to be >20. Thus, for the VAPER Study, creating 1 weight that covers all sociodemographic variables, including gender, age, race, income, and region, is not acceptable as the cell sizes would be under the minimum threshold. Therefore, 3 separate weights are available: a gender, age, and race weight; an income weight; and a geographic region weight. The variables for the gender, age, and race weight are dichotomized as men and women, <35 years and ≥35 years, and White and non-White populations. For the annual income weight, data are dichotomized as US <$60,000 and US ≥$60,000. Finally, for the geographic region weight, data are categorized into “Northeast,” “Midwest,” “South,” and “West.” The specific poststratification weight used in the dissemination of the survey findings will be hypothesis-driven and based on whether the characteristics incorporated into the weight are expected to be correlated highly with the primary outcome of interest.

### Planned Analyses

Analyses will be designed to observe patterns that suggest the benefits and unintended consequences of potential regulations. Our data offer a detailed examination of frequent ENDS and ENNDS users’ device features and specifications, liquid characteristics, and behaviors. A better understanding of how devices and liquids relate to one another and may be associated with key behaviors such as nicotine dependence can provide regulators with a more precise understanding of how regulating features, specifications, and characteristics may positively or negatively affect users before implementing a regulation. We intend to examine these relationships for three potential regulations: (1) limits on nicotine, (2) constraints on nicotine flux (ie, nicotine emitted over time), and (3) reduction in flavor availability (Textbox 1).

**Hypotheses regarding the 3 potential regulations for electronic nicotine delivery systems (ENDS).**

- Limits on nicotine
  As of May 2016, the European Union limits nicotine concentration in liquids; the US Food and Drug Administration (FDA) may consider a similar action. Hypotheses will be informed by the latest available data at the time of analysis, but a priori, we hypothesize that device wattage will be correlated inversely with nicotine concentration and directly with the amount of liquid consumed and that, across concentration, more dependent ENDS users are using higher-power devices. Over time, users who switch to lower-concentration liquids will begin using higher-power devices. This pattern would highlight how limiting liquid nicotine concentration to control nicotine delivery may fail when higher-wattage devices are available.
- Constraints on nicotine flux
  Nicotine flux is a function of device specifications and liquid characteristics (ie, power and nicotine concentration) and helps determine user nicotine exposure; it can be predicted mathematically, suggesting that a flux-based product regulatory standard is possible. We will investigate flux standards by studying device wattage, nicotine concentration, and user dependence. Again, hypotheses will be informed by the latest available data at the time, but a priori, we hypothesize that higher flux conditions (eg, greater wattage and nicotine concentration) will be associated with greater dependence, lower flux conditions will be associated with less dependence, and transitions from lower to high flux conditions will be accompanied by higher flux. This pattern highlights nicotine flux as a potential regulatory target.
- Reduction in flavor availability
  The FDA began prioritizing the enforcement of premarket of ENDS products in 2021 [20,21]. This action has likely decreased the variety of liquid flavors sold. A priori, we hypothesize that this decrease may lead users to change their preferred flavor for their most commonly used device, change their device specifications, or change their behavior to maintain their nicotine delivery. Transition patterns will highlight how reducing flavor availability may affect behavior.

Our primary aims are to evaluate the 3 potential regulations; however, the relative novelty of our methods for collecting detailed device features and specifications and liquid characteristics along with a new practice of analyzing device and liquid pairings rather than analyzing them separately warrants additional supportive and foundational analyses, respectively. As such, our team has identified additional priorities, including but not limited to examining the percentage of agreement between self-reported responses and photo data collection of devices and liquids, mitigating the impact of bots and fraudulent survey takers, and identifying common combinations of device specifications and liquid characteristics (and transitions).

There is no one primary analytic approach; statistical tests will vary based on the research question, measures used, and cross-sectional versus longitudinal nature of the analysis. In general, for longitudinal analyses, generalized estimating equations will be used to account for the variable times between survey waves.

## Discussion

The VAPER Study uses a web-based longitudinal cohort design to observe patterns that suggest the benefits and unintended consequences of 3 potential Food and Drug Administration regulations. A priori, we hypothesize that we will identify relationships among device features and specifications, liquid characteristics, and user behavior. A better understanding of these relationships, particularly longitudinally, may allow regulators to better understand how regulations positively or negatively affect user health.

Most population surveys about ENDS and ENNDS are unable to describe device features and specifications, liquid characteristics, and user behavior in a detailed manner because of measurement-related challenges such as a highly customizable user experience [22] and a lack of standardized reporting requirements for device features and specifications and liquid characteristics [9]. Consequently, surveys often oversimplify use. For instance, surveys sometimes presume that a single device is used and request that participants indicate the device type, usually with predefined definitions that may not keep pace with market innovations (particularly salient for longitudinal surveys) [23,24] or terminology used by all participants [25]. Surveys that use such an approach also ignore the possibility that some users use multiple ENDS and ENNDS in varying amounts and that different device and liquid combinations (within and across device types) may affect nicotine and toxicant emissions and delivery [26]. Our study demonstrates potential solutions to these measurement challenges through survey questions that allow participants to describe their most commonly used device and most commonly used liquid for that device through the use of adaptive questions. In addition, we require valid photos of their most commonly used device and request photos of their most commonly used liquids for that device, thereby allowing our team to determine the device type, features, and specifications and liquid characteristics based on the photo and related website coded data independent of self-reported responses.

Web-based surveys have become increasingly common; however, reporting of contemporary methodologies to maximize data integrity and mitigate the impact of bots and fraudulent survey takers and best practices for de novo recruitment is lacking. Moreover, technology to evade basic survey protections has evolved since the Checklist for Reporting Results of Internet E-Surveys was developed in 2004, and the use of these technologies has become more common. For example, verifying that a sample does not contain duplicative IP addresses and use of “cookies” can be overcome through the use of virtual private network service providers that allow users easy access to hundreds of servers worldwide and the clearing of cookies (or use of another device), respectively. Our survey was able to address these challenges using a variety of strategies, such as identity verification.

Recruitment modalities beyond de novo recruitment were explored and given consideration for this study, including the use of existing panels. Although appropriate for other study designs and aims, existing panels were not considered a viable option for the VAPER Study. Existing panels at well-established research firms did not contain a sample large enough to recruit our population of interest (ie, ENDS and ENNDS users vaping ≥5 days per week). Mechanical Turk, a commonly used web-based panel, was considered as well but was also not large enough for our lower-prevalence population. Panels that aggregate participants from multiple panels are an option that might have yielded a large enough sample for wave 1; however, the recruitment methodologies are highly heterogeneous. In addition, they may not be large enough to replace those lost to follow-up in multiple waves. We were also advised by a company offering this aggregation service that high loss to follow-up rates should be expected; thus, panels that aggregate participants are not ideal for longitudinal studies.

Social media (eg, Facebook and Instagram) recruitment was attempted in consultation with a market research firm at the start of wave 1, but several challenges were encountered. Despite being an academic survey on ENDS and ENNDS use, our advertisements were repeatedly deleted by Facebook and Instagram for including images and text related to ENDS and ENNDS. Appealing these decisions became a regular and time-consuming phenomenon that was never resolved. Furthermore, the advertisements appeared to generate clicks, presumably by social media users interested in the survey; however, the number of advertisement clicks did not match the landing page traffic, and few valid surveys were submitted. Our approach to tracking landing page traffic was investigated for setup errors, and alternative back-end solutions were attempted, but no strategy improved our traffic. The high costs (US $280 per valid participant) combined with slow recruitment (approximately 5 valid participants per week) led us to switch to a Craigslist-focused recruitment strategy. Subsequently, wave 3 costs have decreased to US $10 per valid participant, and recruitment for the baseline survey has increased to approximately 53 valid participants per week.

Processing data and managing missing data are not without challenges. Coding photos and reviewing and abstracting manufacturer, retailer, and review site data are time-intensive and have practical limitations, including participants who do not follow photo submission instructions and inconsistent and incomplete device features and specifications available on the web. The resulting missing data present challenges, particularly given a lack of validated device and liquid questions and that some participants indicate that they do not know the details of their device features and specifications, settings, and liquid characteristics. Our solution is to implement comprehensive preventive and post hoc strategies that maximize the use of multiple data sources (ie, photos, self-report, and disassembled devices), tools (ie, multimeter), and formulas (ie, power calculator and an average midpoint calculation for each device type). The decisions were considered carefully based on the best available information; however, we cannot rule out the possibility that our underlying data sources, tools, or formulas are biased or inaccurate (eg, product packaging and labeling and manufacturer, academic, retailer, and review websites). Ultimately, we believe that the benefits of our approach outweigh the unknowns and are an opportunity to understand more deeply the interplay between devices, liquids, and user behavior as it pertains to regulations. Other strategies could be valuable, and our approach may change as better data sources emerge and more is learned about this topic.

The loss to follow-up rate is higher than initially presumed based on the expected loss to follow-up rates in more traditional cohort surveys. This presumption based on traditional cohort surveys was made after we were unable to find any web-based cohort surveys that reported their loss to follow-up rates during study design planning in 2019. The higher-than-expected rate is not readily explainable and is a matter of speculation. Our assumption is that it is owing to one or more of the following: (1) web-based cohort survey participants require larger incentives, (2) participants (and email providers) may believe that follow-up survey email and SMS text message invitations and reminders are spam, (3) participants may self-select out of the cohort survey if they no longer use e-cigarettes, and (4) participant commitment to web-based cohort surveys is lower given the lack of an in-person connection with the study team. We will attempt to lower the rate through several strategies. These include increasing the incentive to US $30 per participant, sending postcards to participants to remind them of their involvement with the study and our annual raffle, creating a password-protected study website for participants that contains announcements and descriptive data for participants to review, and allowing follow-up participants who no longer use the devices ≥5 days per week to complete the survey or a shorter survey if they have not used the devices in the past 30 days.

Furthermore, our team is committed to improving the survey with each successive data collection wave. We will continue to monitor and adjust the details of our Craigslist strategy, such as the number of cities and boards used, as we learn more about the recruitment and cost-efficiency of “jobs” versus “gigs” postings and specific locations. We are also considering alterations to the raffles based on the frequency of waves completed and participant follow-up rates and are exploring additional engagement strategies to increase retention rates. Other options include birthday and holiday messages, a website containing updates on findings and survey-related announcements, and alternative incentive structures for follow-up survey participants. Moreover, participants are encouraged to provide us with feedback at the end of the survey: “We would like to continually improve this survey. If you have any comments or suggestions about this survey, please provide them here.” Feedback is reviewed during each wave for isolated issues (eg, survey response corrections) and at the end of the survey wave for consistent feedback that may warrant improvements to the survey. For example, participants noted that our survey responses did not include options for wrapping one’s own device coils and adding extra nicotine drops to liquids. We subsequently edited the response options and added a question to account for these behaviors.

Another key lesson learned was the importance of bot and fraudulent survey taker mitigation strategies. These comprehensive steps were taken in consultation with experts after an initial attempt in 2019 to recruit participants using social media advertisements failed. At the time, the VAPER Study allowed for anonymous survey participants, had minimal review of data before incentive delivery by email, and used fraud detection software *intended* to prevent multiple completions by each participant. Initially, recruitment began slowly (as expected) but accelerated quickly, raising substantial concerns that led us to halt data collection. Survey submissions (n=1624) were investigated for evidence of bots and fraudulent survey takers, and only 22.35% (363/1624) of the survey completions were assessed to be likely valid. We subsequently restarted recruitment in May 2020 (wave 1) and implemented the aforementioned risk-mitigation strategy. As a result of these steps and the transition to a Craigslist posting strategy, the recruitment pace stabilized, and data quality has appeared adequate. The final wave 1 sample does not include those from our failed attempt at recruitment and data collection in 2019.

The VAPER Study has demonstrated that web-based recruitment and data collection for cohort studies is a promising approach that may offer benefits to researchers and participants, including convenience, scalability, reduced costs, and safety during extraordinary times such as the COVID-19 pandemic. More specifically, this study design has allowed us to recruit a generalizable and nationwide sample relatively quickly and cheaply, recruit a lower-prevalence population successfully, and disseminate high-level findings to regulators quickly. To the best of our knowledge, no other tobacco policy research team is collecting such detailed data on the scale at which we are operating, which differentiates the value of these data from those of other studies. These data better position us to address questions about the relationships among devices, liquids, and user behavior that relate to the benefits and unintended consequences of possible regulations. However, strong risk-mitigation strategies are essential to ensure data quality [13,14], and steps such as identity verification and manual review of photo data quality before sending the incentive are time-intensive and have required a team of individuals at 2 universities. Other limitations include a limited sample size for specific questions (because of skip logic and rare behaviors), missing data resulting from inconsistent and incomplete device and liquid data available on the web, website-reported data on devices and liquids that may or may not correlate with laboratory-measured device specifications (eg, coil resistance) or liquid characteristics (eg, nicotine concentration), and participants unable to recall or who may be misinformed about their device features and specifications or liquid characteristics. Future plans include further optimizing our recruitment and data processing procedures and conducting 2 waves of data collection over the next 12-month period.

## Appendix

Multimedia Appendix 1 Craigslist posting locations and frequencies for waves 1 to 3.

## Abbreviations

CAPTCHA: Completely Automated Public Turing test to tell Computers and Humans Apart
ENDS: electronic nicotine delivery systems
ENNDS: electronic nonnicotine delivery systems
IRB: institutional review board
REDCap: Research Electronic Data Capture
VAPER: Vaping and Patterns of E-cigarette Use Research

## Data Availability

We intend to make deidentified data, codebooks, documentation, and research protocols available on a password-protected website by 2026 in accordance with the National Institutes of Health policy (notice: NOT-OD-03-032) released on February 26, 2003 [27], and IRB-approved protocols. Before or after the data are available on the aforementioned website, any investigator interested in collaboration or in using the data for their own work is invited to contact the corresponding author. They will be asked to submit a 1-page abstract of their proposed research, including purpose, analytical plan, and dissemination plans. An executive leadership committee will review these proposals and decide on each based on the individual merits (if a proposal is received after the end of the funding period, the principal investigator will review it). Review criteria and prioritization of projects include the potential of the proposed work to advance public health; qualifications of the applicant; potential for publication; potential for future funding; and enhancement of the scientific, geographic, and demographic diversity of the Vaping and Patterns of E-cigarette Use Research Study research portfolio.

## References

[1] Creamer MR, Wang TW, Babb S, Cullen KA, Day H, Willis G, Jamal A, Neff L (2019). Tobacco product use and cessation indicators among adults - United States, 2018. MMWR Morb Mortal Wkly Rep.

[2] Park-Lee E, Ren C, Sawdey MD, Gentzke AS, Cornelius M, Jamal A, Cullen KA (2021). Notes from the field: e-cigarette use among middle and high school students - National Youth Tobacco Survey, United States, 2021. MMWR Morb Mortal Wkly Rep.

[3] (2019). Research Priorities. U.S. Food and Drug Administration.

[4] Eissenberg T, Shihadeh A (2015). Nicotine flux: a potentially important tool for regulating electronic cigarettes. Nicotine Tob Res.

[5] Domino EF (1986). Nicotine: a unique psychoactive drug--arousal with skeletal muscle relaxation. Psychopharmacol Bull.

[6] Rudy AK, Leventhal AM, Goldenson NI, Eissenberg T (2017). Assessing electronic cigarette effects and regulatory impact: challenges with user self-reported device power. Drug Alcohol Depend.

[7] Crespi E, Hardesty JJ, Nian Q, Sinamo J, Welding K, Kennedy RD, Cohen JE (2022). Agreement between self-reports and photos to assess e-cigarette device and liquid characteristics in wave 1 of the vaping and patterns of e-cigarette use research study: web-based longitudinal cohort study. J Med Internet Res.

[8] Taylor A, Dunn K, Turfus S (2021). A review of nicotine-containing electronic cigarettes-trends in use, effects, contents, labelling accuracy and detection methods. Drug Test Anal.

[9] Raymond BH, Collette-Merrill K, Harrison RG, Jarvis S, Rasmussen RJ (2018). The nicotine content of a sample of e-cigarette liquid manufactured in the United States. J Addict Med.

[10] Jackson R, Huskey M, Brown S (2020). Labelling accuracy in low nicotine e-cigarette liquids from a sampling of US manufacturers. Int J Pharm Pract.

[11] Hiler M, Karaoghlanian N, Talih S, Maloney S, Breland A, Shihadeh A, Eissenberg T (2020). Effects of electronic cigarette heating coil resistance and liquid nicotine concentration on user nicotine delivery, heart rate, subjective effects, puff topography, and liquid consumption. Exp Clin Psychopharmacol.

[12] Talih S, Balhas Z, Eissenberg T, Salman R, Karaoghlanian N, El Hellani A, Baalbaki R, Saliba N, Shihadeh A (2015). Effects of user puff topography, device voltage, and liquid nicotine concentration on electronic cigarette nicotine yield: measurements and model predictions. Nicotine Tob Res.

[13] Pozzar R, Hammer MJ, Underhill-Blazey M, Wright AA, Tulsky JA, Hong F, Gundersen DA, Berry DL (2020). Threats of bots and other bad actors to data quality following research participant recruitment through social media: cross-sectional questionnaire. J Med Internet Res.

[14] Guest JL, Adam E, Lucas IL, Chandler CJ, Filipowicz R, Luisi N, Gravens L, Leung K, Chavanduka T, Bonar EE, Bauermeister JA, Stephenson R, Sullivan PS (2021). Methods for authenticating participants in fully web-based mobile app trials from the iReach project: cross-sectional study. JMIR Mhealth Uhealth.

[15] (2018). E-cigarettes: Facts, stats and regulations. Truth Initiative.

[16] Drillinger M (2022). E-cig on the rise among middle and high school students. Healthline.

[17] Fagan P, Pohkrel P, Herzog T, Pagano I, Vallone D, Trinidad DR, Sakuma KL, Sterling K, Fryer CS, Moolchan E (2015). Comparisons of three nicotine dependence scales in a multiethnic sample of young adult menthol and non-menthol smokers. Drug Alcohol Depend.

[18] Freisthler B, Gruenewald PJ, Tebben E, Shockley McCarthy K, Price Wolf J (2021). Understanding at-the-moment stress for parents during COVID-19 stay-at-home restrictions. Soc Sci Med.

[19] (2022). Article Resource Page. Institute for Global Tobacco Control.

[20] (2016). Deeming Tobacco Products To Be Subject to the Federal Food, Drug, and Cosmetic Act, as Amended by the Family Smoking Prevention and Tobacco Control Act; Restrictions on the Sale and Distribution of Tobacco Products and Required Warning Statements for Tobacco Products. The Daily Journal of the United States Government.

[21] Hahn SM (2020). Coronavirus (COVID-19) update: court grants FDA’s request for extension of premarket review submission deadline for certain tobacco products because of impacts from COVID-19. U.S. Food and Drug Administration.

[22] Soule E, Bansal-Travers M, Grana R, McIntosh S, Price S, Unger JB, Walton K (2023). Electronic cigarette use intensity measurement challenges and regulatory implications. Tob Control.

[23] Weaver SR, Kim H, Glasser AM, Sutfin EL, Barrington-Trimis J, Payne TJ, Saddleson M, Loukas A (2018). Establishing consensus on survey measures for electronic nicotine and non-nicotine delivery system use: current challenges and considerations for researchers. Addict Behav.

[24] O'Connor R, Durkin SJ, Cohen JE, Barnoya J, Henriksen L, Hill SE, Malone RE (2021). Thoughts on neologisms and pleonasm in scientific discourse and tobacco control. Tob Control.

[25] Alexander JP, Coleman BN, Johnson SE, Tessman GK, Tworek C, Dickinson DM (2016). Smoke and vapor: exploring the terminology landscape among electronic cigarette users. Tob Regul Sci.

[26] El-Hellani A, Salman R, El-Hage R, Talih S, Malek N, Baalbaki R, Karaoghlanian N, Nakkash R, Shihadeh A, Saliba NA (2018). Nicotine and carbonyl emissions from popular electronic cigarette products: correlation to liquid composition and design characteristics. Nicotine Tob Res.

[27] (2003). Final NIH statement on sharing research data. National Institutes of Health (NIH).

