# Supplementary material for: The Vaping and Patterns of e-Cigarette Use Research Study: Protocol for a Web-Based Cohort Study
Source: JMIR Res Protoc. 2023 Mar 2;12:e38732. doi: 10.2196/38732 (PMC10020901; doi:10.2196/38732)
Supplement: Multimedia Appendix 1 [file resprot_v12i1e38732_app1.docx]

| **Location** | **State** | **Location, State** | **Location Code** | **Region** | **Posted in W1** | **Posted in W2** | **Posted in W3** | **Frequency of posting in W3** |
| --- | --- | --- | --- | --- | --- | --- | --- | --- |
| Abilene | TX | Abilene TX | ABI | South | N | N | Y | Monthly |
| Akron | OH | Akron OH | CAK | Midwest | Y | Y | Y | Monthly |
| Albany | GA | Albany GA | ABY | South | N | N | Y | Monthly |
| Albany | NY | Albany NY | ALB | Northeast | N | N | Y | Monthly |
| Albuquerque | NM | Albuquerque NM | ABQ | West | Y | Y | Y | Every two weeks |
| Altoona-Johnstown | PA | Altoona-Johnstown PA | AOO | Northeast | N | N | Y | Monthly |
| Amarillo | TX | Amarillo TX | AMA | South | Y | Y | Y | Monthly |
| Ames | IA | Ames IA | AME | Midwest | N | N | Y | Monthly |
| Anchorage | AK | Anchorage AK | ANC | West | Y | Y | Y | Monthly |
| Ann Arbor | MI | Ann Arbor MI | AAA | Midwest | N | N | Y | Monthly |
| Annapolis | MD | Annapolis MD | ANP | South | N | N | Y | Monthly |
| Appleton-Oshkosh-FDL | WI | Appleton-Oshkosh-FDL WI | APP | Midwest | N | N | Y | Monthly |
| Asheville | NC | Asheville NC | ASH | South | N | N | Y | Monthly |
| Ashtabula | OH | Ashtabula OH | JFN | Midwest | N | N | Y | Monthly |
| Athens | GA | Athens GA | AHN | South | N | N | Y | Monthly |
| Athens OH | OH | Athens OH | OHU | Midwest | N | N | Y | Monthly |
| Atlanta | GA | Atlanta GA | ATL - ATL | South | Y | Y | Y | Twice a week |
| Auburn | AL | Auburn AL | AUB | South | N | N | Y | Monthly |
| Augusta | GA | Augusta GA | AUG | South | Y | Y | Y | Monthly |
| Austin | TX | Austin TX | AUS | South | Y | Y | Y | Twice a week |
| Bakersfield | CA | Bakersfield CA | BAK | West | Y | Y | Y | Monthly |
| Baltimore | MD | Baltimore MD | BAL | South | Y | Y | Y | Weekly |
| Baton Rouge | LA | Baton Rouge LA | BTR | South | Y | Y | Y | Monthly |
| Battle Creek | MI | Battle Creek MI | BTC | Midwest | N | N | Y | Monthly |
| Beaumont/Port Arthur | TX | Beaumont/Port Arthur TX | BPT | South | N | N | Y | Monthly |
| Bellingham | WA | Bellingham WA | BLI | West | N | N | Y | Monthly |
| Bemidji | MN | Bemidji MN | BJI | Midwest | N | N | Y | Monthly |
| Bend | OR | Bend OR | BND | West | N | N | Y | Monthly |
| Billings | MT | Billings MT | BIL | West | Y | Y | Y | Monthly |
| Binghamton | NY | Binghamton NY | BGM | Northeast | N | N | Y | Monthly |
| Birmingham | AL | Birmingham AL | BHM | South | Y | Y | Y | Monthly |
| Bismarck | ND | Bismarck ND | BIS | Midwest | N | N | Y | Monthly |
| Bloomington | IL | Bloomington IL | BMG | Midwest | N | N | Y | Monthly |
| Bloomington-Normal | IL | Bloomington-Normal IL | BLN | Midwest | N | N | Y | Monthly |
| Boise | ID | Boise ID | BOI | West | Y | Y | Y | Every two weeks |
| Boone | NC | Boone NC | BNC | South | N | N | Y | Monthly |
| Boston | MA | Boston MA | BOS - GBS | Northeast | Y | Y | Y | Twice a week |
| Boulder | CO | Boulder CO | BOU | West | N | N | Y | Monthly |
| Bowling Green | KY | Bowling Green KY | BLG | South | N | N | Y | Monthly |
| Bozeman | MT | Bozeman MT | BZN | West | N | N | Y | Monthly |
| Brainerd | MN | Brainerd MN | BRD | Midwest | N | N | Y | Monthly |
| Bridgeport | CT | Bridgeport CT | NLO | Northeast | Y | Y | Y | Monthly |
| Brownsville | TX | Brownsville TX | BRO | South | Y | Y | Y | Monthly |
| Brunswick | GA | Brunswick GA | BWK | South | N | N | Y | Monthly |
| Buffalo | NY | Buffalo NY | BUF | Northeast | Y | Y | Y | Monthly |
| Butte | MT | Butte MT | BTM | West | N | N | Y | Monthly |
| Cape Cod/Islands | MA | Cape Cod/Islands MA | CAP | Northeast | N | N | Y | Monthly |
| Catskills | NY | Catskills NY | CAT | Northeast | N | N | Y | Monthly |
| Cedar Rapids | IA | Cedar Rapids IA | CED | Midwest | N | N | Y | Monthly |
| Central Louisiana | LA | Central Louisiana LA | AEX | South | N | N | Y | Monthly |
| Central Michigan | MI | Central Michigan MI | CMU | Midwest | N | N | Y | Monthly |
| Central NJ | NJ | Central NJ | CNJ | Northeast | N | N | Y | Twice a week |
| Champaign Urbana | IL | Champaign Urbana IL | CHM | Midwest | N | N | Y | Monthly |
| Charleston | SC | Charleston SC | CHS | South | Y | Y | Y | Monthly |
| Charleston | WV | Charleston WV | CRW | South | Y | Y | Y | Monthly |
| Charlotte | NC | Charlotte NC | CHA | South | Y | Y | Y | Weekly |
| Charlottesville | VA | Charlottesville VA | UVA | South | N | N | Y | Monthly |
| Chattanooga | TN | Chattanooga TN | CHT | South | Y | Y | Y | Monthly |
| Chautauqua | NY | Chautauqua NY | CHQ | Northeast | N | N | Y | Monthly |
| Chicago | IL | Chicago IL | CHI - CHC | Midwest | Y | Y | Y | Twice a week |
| Chico | CA | Chico CA | CHC | West | N | N | Y | Monthly |
| Chillicothe | OH | Chillicothe OH | CHL | Midwest | N | N | Y | Monthly |
| Cincinnati | OH | Cincinnati OH | CIN | Midwest | Y | Y | Y | Every two weeks |
| Clarksville | TN | Clarksville TN | CKV | South | Y | Y | Y | Monthly |
| Cleveland | OH | Cleveland OH | CLE | Midwest | Y | Y | Y | Every two weeks |
| Clovis/Portales | NM | Clovis/Portales NM | CVN | West | N | N | Y | Monthly |
| College Station | TX | College Station TX | CST | South | N | N | Y | Monthly |
| Colorado Springs | CO | Colorado Springs CO | COS | West | Y | Y | Y | Every two weeks |
| Columbia | SC | Columbia SC | CAE | South | N | N | Y | Monthly |
| Columbia/Jeff City | MO | Columbia/Jeff City MO | COU | Midwest | N | N | Y | Monthly |
| Columbus | OH | Columbus OH | COL | Midwest | Y | Y | Y | Weekly |
| Columbus | GA | Columbus GA | CSG | South | Y | Y | N | - |
| Cookeville | TN | Cookeville TN | COO | South | N | N | Y | Monthly |
| Corpus Christi | TX | Corpus Christi TX | CRP | South | Y | Y | Y | Monthly |
| Corvallis/Albany | OR | Corvallis/Albany OR | CRV | West | N | N | Y | Monthly |
| Cumberland Valley | PA | Cumberland Valley PA | CBG | Northeast | N | N | Y | Monthly |
| Dallas | TX | Dallas TX | DAL - DAL | South | Y | Y | Y | Twice a week |
| Danville | VA | Danville VA | DNV | South | N | N | Y | Monthly |
| Dayton | OH | Dayton OH | DAY | Midwest | Y | Y | Y | Monthly |
| Daytona Beach | FL | Daytona Beach FL | DAB | South | N | N | Y | Monthly |
| Decatur | IL | Decatur IL | DIL | Midwest | N | N | Y | Monthly |
| Deep East Texas | TX | Deep East Texas TX | OCH | South | N | N | Y | Monthly |
| Del Rio/Eagle Pass | TX | Del Rio/Eagle Pass TX | DRT | South | N | N | Y | Monthly |
| Delaware (State) | DE | Delaware (state) | DLW | South | Y | Y | Y | Monthly |
| Denver | CO | Denver CO | DEN | West | Y | Y | Y | Weekly |
| Des Moines | IA | Des Moines IA | DSM | Midwest | Y | Y | Y | Monthly |
| Detroit | MI | Detroit MI | DET - WYN | Midwest | Y | Y | Y | Weekly |
| Dothan | AL | Dothan AL | DHN | South | N | N | Y | Monthly |
| Dubuque | IA | Dubuque IA | DBQ | Midwest | N | N | Y | Monthly |
| Duluth/Superior | MN | Duluth/Superior MN | DLH | Midwest | N | N | Y | Monthly |
| East Idaho | ID | East Idaho ID | EID | West | N | N | Y | Monthly |
| East Oregon | OR | East Oregon OR | EOR | West | N | N | Y | Monthly |
| Eastern CO | CO | Eastern CO | ECO | West | N | N | Y | Monthly |
| Eastern Kentucky | KY | Eastern Kentucky KY | EKY | South | N | N | Y | Monthly |
| Eastern Montana | MT | Eastern Montana MT | MNT | West | N | N | Y | Monthly |
| Eastern NC | NC | Eastern NC | ENC | South | N | N | Y | Monthly |
| Eastern Panhandle | WV | Eastern Panhandle WV | EWV | South | N | N | Y | Monthly |
| Eastern Shore | MD | Eastern Shore MD | ESH | South | N | N | Y | Monthly |
| Eau Claire | WI | Eau Claire WI | EAU | Midwest | N | N | Y | Monthly |
| El Paso | TX | El Paso TX | ELP | South | Y | Y | Y | Monthly |
| Elko | NV | Elko NV | ELK | West | N | N | Y | Monthly |
| Elmira-Corning | NY | Elmira-Corning NY | ELM | Northeast | N | N | Y | Monthly |
| Erie | PA | Erie PA | ERI | Northeast | N | N | Y | Monthly |
| Eugene | OR | Eugene OR | EUG | West | Y | Y | Y | Monthly |
| Evansville | IN | Evansville IN | EW | Midwest | N | N | Y | Monthly |
| Fairbanks | AK | Fairbanks AK | FAI | West | N | N | Y | Monthly |
| Fargo/Moorhead | ND | Fargo/Moorhead ND | FAR | Midwest | N | N | Y | Monthly |
| Farmington | NM | Farmington NM | FNM | West | N | N | Y | Monthly |
| Fayetteville | AR | Fayetteville AR | FYV | South | N | N | Y | Monthly |
| Fayetteville | NC | Fayetteville NC | FAY | South | Y | Y | Y | Monthly |
| Finger Lakes | NY | Finger Lakes NY | FGL | Northeast | N | N | Y | Monthly |
| Flagstaff/Sedona | AZ | Flagstaff/Sedona AZ | FLG | West | N | N | Y | Monthly |
| Flint | MI | Flint MI | FNT | Midwest | N | N | Y | Monthly |
| Florence/Muscle Shoals | AL | Florence/Muscle Shoals AL | MSL | South | N | N | Y | Monthly |
| Florida Keys | FL | Florida Keys FL | KEY | South | N | N | Y | Monthly |
| Fort Collins/North | CO | Fort Collins/North CO | FTC | West | N | N | Y | Monthly |
| Fort Dodge | IA | Fort Dodge IA | FTD | Midwest | N | N | Y | Monthly |
| Fort Smith | AR | Fort Smith AR | FSM | South | N | N | Y | Monthly |
| Frederick | MD | Frederick MD | FDK | South | N | N | Y | Monthly |
| Fredericksburg | VA | Fredericksburg VA | EZF | South | N | N | Y | Monthly |
| Fresno | CA | Fresno CA | FRE | West | Y | Y | Y | Monthly |
| Ft Myers | FL | Ft Myers FL | FMY - LEE | South | Y | Y | Y | Monthly |
| Ft Wayne | IN | Ft Wayne IN | FWA | Midwest | Y | Y | Y | Monthly |
| Gadsden-Anniston | AL | Gadsden-Anniston AL | ANB | South | N | N | Y | Monthly |
| Galveston | TX | Galveston TX | GLS | South | N | N | Y | Twice a week |
| Glens Falls | NY | Glens Falls NY | GFL | Northeast | N | N | Y | Monthly |
| Gold Country | CA | Gold Country CA | GLD | West | N | N | Y | Monthly |
| Grand Forks | ND | Grand Forks ND | GFK | Midwest | N | N | Y | Monthly |
| Grand Island | NE | Grand Island NE | GIL | Midwest | N | N | Y | Monthly |
| Grand Rapids | MI | Grand Rapids MI | GRR | Midwest | Y | Y | Y | Monthly |
| Great Falls | MT | Great Falls MT | GTF | West | N | N | Y | Monthly |
| Green Bay | WI | Green Bay WI | GRB | Midwest | N | N | Y | Monthly |
| Greensboro | NC | Greensboro NC | GBO | South | Y | Y | Y | Every two weeks |
| Greenville/Upstate | SC | Greenville/Upstate SC | GSP | South | N | N | Y | Monthly |
| Gulfport/Biloxi | CA | Gulfport/Biloxi CA | GPT | West | N | N | Y | Monthly |
| Hanford-Corcoran | CA | Hanford-Corcoran CA | HNF | West | N | N | Y | Monthly |
| Harrisburg | PA | Harrisburg PA | HRS | Northeast | N | N | Y | Monthly |
| Harrisonburg | VA | Harrisonburg VA | SHD | South | N | N | Y | Monthly |
| Hartford | CT | Hartford CT | HTF | Northeast | N | N | Y | Monthly |
| Hattiesburg | MS | Hattiesburg MS | USM | South | N | N | Y | Monthly |
| Heartland Florida | FL | Heartland Florida FL | CFL | South | N | N | Y | Monthly |
| Helena | MT | Helena MT | HLN | West | N | N | Y | Monthly |
| Hickory/Lenoir | SC | Hickory/Lenoir SC | HKY | South | N | N | Y | Monthly |
| High Rockies | CO | High Rockies CO | RCK | West | N | N | Y | Monthly |
| Hilton Head | SC | Hilton Head SC | HHI | South | N | N | Y | Monthly |
| Holland | MI | Holland MI | HLD | Midwest | N | N | Y | Monthly |
| Honolulu | HI | Honolulu HI | HNL - OAH | West | Y | Y | Y | Weekly |
| Houma | LA | Houma LA | HUM | South | N | N | Y | Monthly |
| Houston | TX | Houston TX | HOU | South | Y | Y | Y | Twice a week |
| Hudson Valley | NY | Hudson Valley NY | HUD | Northeast | N | N | Y | Monthly |
| Humboldt County | CA | Humboldt County CA | HMB | West | N | N | Y | Monthly |
| Huntington-Ashland | WV | Huntington-Ashland WV | HTS | South | N | N | Y | Monthly |
| Huntsville | AL | Huntsville AL | HSV | South | Y | Y | Y | Monthly |
| Imperial County | CA | Imperial County CA | IMP | West | N | N | Y | Monthly |
| Indianapolis | IN | Indianapolis IN | IND | Midwest | Y | Y | Y | Every two weeks |
| Iowa City | IA | Iowa City IA | IAC | Midwest | N | N | Y | Monthly |
| Ithaca | NY | Ithaca NY | ITH | Northeast | N | N | Y | Monthly |
| Jackson | MI | Jackson MI | JXN | South | N | N | Y | Monthly |
| Jackson | TN | Jackson TN | JXT | South | N | N | Y | Monthly |
| Jackson | MS | Jackson MS | JAN | South | Y | Y | Y | Monthly |
| Jacksonville | NC | Jacksonville NC | OAJ | South | N | N | Y | Monthly |
| Jacksonville | FL | Jacksonville FL | JAX | South | Y | Y | Y | Weekly |
| Jersey Shore | NJ | Jersey Shore NJ | JYS | Northeast | N | N | Y | Monthly |
| Jonesboro | AR | Jonesboro AR | JBR | South | N | N | Y | Monthly |
| Joplin | MO | Joplin MO | JLN | Midwest | N | N | Y | Monthly |
| Kalamazoo | MI | Kalamazoo MI | KZO | Midwest | N | N | Y | Monthly |
| Kalispell | MT | Kalispell MT | FCA | West | N | N | Y | Monthly |
| Kansas City | MO | Kansas City MO | KSC | Midwest | Y | Y | Y | Every two weeks |
| Kenai Peninsula | AK | Kenai Peninsula AK | ENA | West | N | N | Y | Monthly |
| Kennewick-Pasco-Richland | WA | Kennewick-Pasco-Richland WA | KPR | West | N | N | Y | Monthly |
| Kenosha-Racine | WI | Kenosha-Racine WI | RAC | Midwest | N | N | Y | Monthly |
| Killeen | TX | Killeen TX | GRK | South | Y | Y | Y | Monthly |
| Kirksville | MO | Kirksville MO | KRK | Midwest | N | N | Y | Monthly |
| Klamath Falls | OR | Klamath Falls OR | KLF | West | N | N | Y | Monthly |
| Knoxville | TN | Knoxville TN | KNX | South | Y | Y | Y | Monthly |
| Kokomo | IN | Kokomo IN | OKK | Midwest | N | N | Y | Monthly |
| La Crosse | WI | La Crosse WI | LSE | Midwest | N | N | Y | Monthly |
| La Salle | CO | La Salle CO | LSL | West | N | N | Y | Monthly |
| Lafayette | LA | Lafayette LA | LFT | South | N | N | Y | Monthly |
| Lafayette/West Lafayette | IN | Lafayette/West Lafayette IN | LAF | Midwest | N | N | Y | Monthly |
| Lake Charles | LA | Lake Charles LA | LKC | South | N | N | Y | Monthly |
| Lake Of The Ozarks | MO | Lake Of The Ozarks MO | LOZ | Midwest | N | N | Y | Monthly |
| Lakeland | FL | Lakeland FL | LAL | South | N | N | Y | Monthly |
| Lancaster | PA | Lancaster PA | LNS | Northeast | N | N | Y | Monthly |
| Lansing | MI | Lansing MI | LAN | Midwest | N | N | Y | Monthly |
| Laredo | TX | Laredo TX | LRD | South | Y | Y | Y | Monthly |
| Las Cruces | NM | Las Cruces NM | LCR | West | N | N | Y | Monthly |
| Las Vegas | NV | Las Vegas NV | LVG | West | Y | Y | Y | Twice a week |
| Lawrence | KS | Lawrence KS | LWR | Midwest | N | N | Y | Monthly |
| Lawton | OK | Lawton OK | LAW | South | N | N | Y | Monthly |
| Lehigh Valley | PA | Lehigh Valley PA | ALT | Northeast | N | N | Y | Monthly |
| Lewiston/Clarkston | ID | Lewiston/Clarkston ID | LWS | West | N | N | Y | Monthly |
| Lexington | KY | Lexington KY | LEX | South | Y | Y | Y | Monthly |
| Lima/Findlay | OH | Lima/Findlay OH | LMA | Midwest | N | N | Y | Monthly |
| Lincoln | NE | Lincoln NE | LNK | Midwest | Y | Y | Y | Monthly |
| Little Rock | AR | Little Rock AR | LIT | South | Y | Y | Y | Monthly |
| Logan | UT | Logan UT | LGU | West | N | N | Y | Monthly |
| Los Angeles | CA | Los Angeles CA | LAX - LAC | West | Y | Y | Y | Twice a week |
| Louisville | KY | Louisville KY | LOU | South | Y | Y | Y | Monthly |
| Lubbock | TX | Lubbock TX | LBB | South | Y | Y | Y | Monthly |
| Lynchburg | VA | Lynchburg VA | LYN | South | N | N | Y | Monthly |
| Macon | GA | Macon GA | MCN | South | Y | Y | Y | Monthly |
| Madison | WI | Madison WI | MAD | Midwest | Y | Y | Y | Monthly |
| Maine (State) | ME | Maine (State) | MNE | Northeast | Y | Y | Y | Monthly |
| Manhattan | KS | Manhattan KS | MHK | Midwest | N | N | Y | Monthly |
| Mankato | MN | Mankato MN | MKT | Midwest | N | N | Y | Monthly |
| Mansfield | OH | Mansfield OH | MFD | Midwest | N | N | Y | Monthly |
| Mason City | IA | Mason City IA | MSC | Midwest | N | N | Y | Monthly |
| Mattoon-Charleston | IL | Mattoon-Charleston IL | MTO | Midwest | N | N | Y | Monthly |
| Mcallen | TX | Mcallen TX | MCA | South | Y | Y | Y | Monthly |
| Meadville | PA | Meadville PA | MDV | Northeast | N | N | Y | Monthly |
| Medford-Ashland | OR | Medford-Ashland OR | MFR | West | N | N | Y | Monthly |
| Memphis | TN | Memphis TN | MEM | South | Y | Y | Y | Every two weeks |
| Mendocino County | CA | Mendocino County CA | MDO | West | N | N | Y | Monthly |
| Merced | CA | Merced CA | MER | West | N | N | Y | Monthly |
| Meridian | MS | Meridian MS | MEI | South | N | N | Y | Monthly |
| Miami | FL | Miami FL | MIA - MDC | South | Y | Y | Y | Twice a week |
| Midland | TX | Midland TX | ODM | South | Y | Y | Y | Monthly |
| Milwaukee | WI | Milwaukee WI | MIL | Midwest | Y | Y | Y | Every two weeks |
| Minneapolis | MN | Minneapolis MN | MIN - HNP | Midwest | Y | Y | Y | Twice a week |
| Missoula | MT | Missoula MT | MSO | West | Y | Y | Y | Monthly |
| Mobile | AL | Mobile AL | MOB | South | Y | Y | Y | Monthly |
| Modesto | CA | Modesto CA | MOD | West | Y | Y | Y | Monthly |
| Mohave County | AZ | Mohave County AZ | MHV | West | N | N | Y | Monthly |
| Monroe | LA | Monroe LA | MLU | South | N | N | Y | Monthly |
| Monroe | MI | Monroe MI | MNR | Midwest | N | N | Y | Monthly |
| Monterey Bay (Salinas) | CA | Monterey Bay (Salinas) CA | MTB | West | Y | Y | Y | Monthly |
| Montgomery | AL | Montgomery AL | MGM | South | Y | Y | Y | Monthly |
| Morgantown | WV | Morgantown WV | WVU | South | Y | Y | Y | Monthly |
| Muncie/Anderson | IN | Muncie/Anderson IN | MUN | Midwest | N | N | Y | Monthly |
| Muskegon | MI | Muskegon MI | MKG | Midwest | N | N | Y | Monthly |
| Myrtle Beach | SC | Myrtle Beach SC | MYR | South | N | N | Y | Monthly |
| Nashville | TN | Nashville TN | NSH | South | Y | Y | Y | Weekly |
| New Hampshire (State) | NH | New Hampshire (state) | NHM | Northeast | Y | Y | Y | Every two weeks |
| New Haven | CT | New Haven CT | HVN | Northeast | N | N | Y | Monthly |
| New Orleans | LA | New Orleans LA | NOR | South | Y | Y | Y | Weekly |
| New River Valley | VA | New River Valley VA | VPI | South | N | N | Y | Monthly |
| New York City | NY | New York City NY | NYC - MNH | Northeast | Y | Y | Y | Twice a week |
| Newark (North Jersey) | NJ | Newark (North Jersey) NJ | NJY | Northeast | Y | Y | Y | Twice a week |
| Norfolk | VA | Norfolk VA | NFK | South | Y | Y | Y | Every two weeks |
| North Central | FL | North Central FL | LCQ | South | N | N | Y | Monthly |
| North Dakota (State) | ND | North Dakota (state) | NDK | Midwest | Y | Y | Y | Monthly |
| North Mississippi | MS | North Mississippi MS | NMS | South | N | N | Y | Monthly |
| North Platte | NE | North Platte NE | LBF | Midwest | N | N | Y | Monthly |
| Northeast SD | SD | Northeast SD | ABR | Midwest | N | N | Y | Monthly |
| Northern Michigan | MI | Northern Michigan MI | NMI | Midwest | N | N | Y | Monthly |
| Northern Panhandle | WV | Northern Panhandle WV | WHL | South | N | N | Y | Monthly |
| Northwest GA | GA | Northwest GA | NWG | South | N | N | Y | Monthly |
| Northwest CT | CT | Northwest CT | NCT | Northeast | N | N | Y | Monthly |
| Northwest KS | KS | Northwest KS | NWK | Midwest | N | N | Y | Monthly |
| Northwest OK | OK | Northwest OK | END | South | N | N | Y | Monthly |
| Ogden-Clearfield | UT | Ogden-Clearfield UT | OGD | West | N | N | Y | Monthly |
| Okaloosa/Walton | FL | Okaloosa/Walton FL | VPS | South | N | N | Y | Monthly |
| Oklahoma City | OK | Oklahoma City OK | OKC | South | Y | Y | Y | Every two weeks |
| Olympic Peninsula | WA | Olympic Peninsula WA | OLP | West | N | N | Y | Monthly |
| Omaha | NE | Omaha NE | OMA | Midwest | Y | Y | Y | Every two weeks |
| Oneonta | NY | Oneonta NY | ONH | Northeast | N | N | Y | Monthly |
| Orange County | CA | Orange County CA | ORC | West | Y | Y | Y | Weekly |
| Oregon Coast | OR | Oregon Coast OR | COR | West | N | N | Y | Monthly |
| Orlando | FL | Orlando FL | ORL | South | Y | Y | Y | Twice a week |
| Outer Banks | NC | Outer Banks NC | OBX | South | N | N | Y | Monthly |
| Owensboro | KY | Owensboro KY | OWB | South | N | N | Y | Monthly |
| Palm Springs | CA | Palm Springs CA | PSP | West | N | N | Y | Monthly |
| Panama City | FL | Panama City FL | PFN | South | N | N | Y | Monthly |
| Parkersburg-Marietta | WV | Parkersburg-Marietta WV | PKB | South | N | N | Y | Monthly |
| Pensacola | FL | Pensacola FL | PNS | South | N | N | Y | Monthly |
| Peoria | IL | Peoria IL | PIA | Midwest | N | N | Y | Monthly |
| Philadelphia | PA | Philadelphia PA | PHI | Northeast | Y | Y | Y | Twice a week |
| Phoenix | AZ | Phoenix AZ | PHX - CPH | West | Y | Y | Y | Twice a week |
| Pierre/Central | SD | Pierre/Central SD | CSD | Midwest | N | N | Y | Monthly |
| Pittsburgh | PA | Pittsburgh PA | PIT | Northeast | Y | Y | Y | Weekly |
| Plattsburgh-Adirondacks | NY | Plattsburgh-Adirondacks NY | PLB | Northeast | N | N | Y | Monthly |
| Poconos | PA | Poconos PA | POC | Northeast | N | N | Y | Monthly |
| Port Huron | MI | Port Huron MI | PHN | Midwest | N | N | Y | Monthly |
| Portland | OR | Portland OR | PDX - MLT | West | Y | Y | Y | Twice a week |
| Potsdam-Canton-Massena | NY | Potsdam-Canton-Massena NY | PTD | Northeast | N | N | Y | Monthly |
| Prescott | AZ | Prescott AZ | PRC | West | N | N | Y | Monthly |
| Providence | RI | Providence RI | PRV | Northeast | Y | Y | Y | Every two weeks |
| Provo/Orem | UT | Provo/Orem UT | PVU | West | N | N | Y | Monthly |
| Pueblo | CO | Pueblo CO | PUB | West | N | N | Y | Monthly |
| Pullman / Moscow | WA | Pullman / Moscow WA | PLM | West | N | N | Y | Monthly |
| Quad Cities | IA | Quad Cities IA | MLI | Midwest | N | N | Y | Monthly |
| Raleigh | NC | Raleigh NC | RAL | South | Y | Y | Y | Weekly |
| Rapid City/West | SD | Rapid City/West SD | RAP | Midwest | N | N | Y | Monthly |
| Reading | PA | Reading PA | REA | Northeast | N | N | Y | Monthly |
| Redding | CA | Redding CA | RDD | West | N | N | Y | Monthly |
| Reno | NV | Reno NV | RNO | West | Y | Y | Y | Every two weeks |
| Richmond | IN | Richmond IN | RIN | Midwest | N | N | Y | Monthly |
| Richmond | VA | Richmond VA | RIC | South | Y | Y | Y | Every two weeks |
| Riverside/Inland Empire | CA | Riverside/Inland Empire CA | INL | West | Y | Y | Y | Weekly |
| Roanoke | VA | Roanoke VA | ROA | South | N | N | Y | Monthly |
| Rochester | MN | Rochester MN | RMN | South | N | N | Y | Monthly |
| Rochester | NY | Rochester NY | RCS | Northeast | Y | Y | Y | Monthly |
| Rockford | IL | Rockford IL | RFD | Midwest | Y | Y | Y | Monthly |
| Roseburg | OR | Roseburg OR | RBG | West | N | N | Y | Monthly |
| Roswell/Carlsbad | NM | Roswell/Carlsbad NM | ROW | West | N | N | Y | Monthly |
| Sacramento | CA | Sacramento CA | SAC | West | Y | Y | Y | Weekly |
| Saginaw-Midland-Baycity | MI | Saginaw-Midland-Baycity MI | MBS | Midwest | N | N | Y | Monthly |
| Salem | OR | Salem OR | SLE | West | Y | Y | Y | Monthly |
| Salina | KS | Salina KS | SNS | Midwest | N | N | Y | Monthly |
| Salt Lake City | UT | Salt Lake City UT | SLC | West | N | N | Y | Monthly |
| San Angelo | TX | San Angelo TX | SJT | South | N | N | Y | Monthly |
| San Antonio | TX | San Antonio TX | SAT | South | Y | Y | Y | Twice a week |
| San Diego | CA | San Diego CA | SDO - CSD | West | Y | Y | Y | Twice a week |
| San Francisco | CA | San Francisco CA | SFO - SFC | West | Y | Y | Y | Twice a week |
| San Luis Obispo | CA | San Luis Obispo CA | SLO | West | N | N | Y | Monthly |
| San Marcos | TX | San Marcos TX | TSU | South | N | N | Y | Monthly |
| Sandusky | OH | Sandusky OH | SKY | Midwest | N | N | Y | Monthly |
| Santa Barbara | CA | Santa Barbara CA | SBA | West | N | N | Y | Monthly |
| Santa Fe/Taos | NM | Santa Fe/Taos NM | SAF | West | N | N | Y | Monthly |
| Sarasota-Bradenton | FL | Sarasota-Bradenton FL | SRQ | South | N | N | Y | Monthly |
| Savannah | GA | Savannah GA | SAV | South | Y | Y | Y | Monthly |
| Scottsbluff/Panhandle | NE | Scottsbluff/Panhandle NE | BFF | Midwest | N | N | Y | Monthly |
| Scranton/Wilkes-Barre | PA | Scranton/Wilkes-Barre PA | AVP | Northeast | N | N | Y | Monthly |
| Seattle | WA | Seattle WA | SEA - SEE | West | Y | Y | Y | Twice a week |
| Sheboygan | WI | Sheboygan WI | SBM | Midwest | N | N | Y | Monthly |
| Show Low | AZ | Show Low AZ | SOW | West | N | N | Y | Monthly |
| Shreveport | LA | Shreveport LA | SHV | South | Y | Y | Y | Monthly |
| Sierra Vista | AZ | Sierra Vista AZ | FHU | West | N | N | Y | Monthly |
| Sioux City | IA | Sioux City IA | SUX | Midwest | N | N | Y | Monthly |
| Sioux Falls | SD | Sioux Falls SD | FSD | Midwest | Y | Y | Y | Monthly |
| Siskiyou County | CA | Siskiyou County CA | SSK | West | N | N | Y | Monthly |
| Skagit/Island/Sji | WA | Skagit/Island/Sji WA | MVW | West | N | N | Y | Monthly |
| South Bend/Michiana | IN | South Bend/Michiana IN | SBN | Midwest | N | N | Y | Monthly |
| South Coast | MA | South Coast MA | SMA | Northeast | N | N | Y | Monthly |
| South Dakota | SD | South Dakota SD | SDK | Midwest | N | N | Y | Monthly |
| South Jersey | NJ | South Jersey NJ | SNJ | Northeast | N | N | Y | Monthly |
| Southeast Alaska | AK | Southeast Alaska AK | JNU | West | N | N | Y | Monthly |
| Southeast IA | IA | Southeast IA | OTU | Midwest | N | N | Y | Monthly |
| Southeast KS | KS | Southeast KS | SEK | Midwest | N | N | Y | Monthly |
| Southeast Missouri | MO | Southeast MO | SMO | Midwest | N | N | Y | Monthly |
| Southern | WV | Southern WV | SWV | South | N | N | Y | Monthly |
| Southern Illinois | IL | Southern Illinois IL | CBD | Midwest | N | N | Y | Monthly |
| Southern Maryland | MD | Southern Maryland MD | SMD | South | N | N | Y | Monthly |
| Southwest KS | KS | Southwest KS | SWK | Midwest | N | N | Y | Monthly |
| Southwest Michigan | MI | Southwest MI | SWM | Midwest | N | N | Y | Monthly |
| Southwest MN | MN | Southwest MN | MML | Midwest | N | N | Y | Monthly |
| Southwest MS | MS | Southwest MS | HEZ | South | N | N | Y | Monthly |
| Southwest TX | TX | Southwest TX | WTX | South | N | N | Y | Monthly |
| Southwest VA | VA | Southwest VA | VAW | South | N | N | Y | Monthly |
| Space Coast | FL | Space Coast FL | MLB | South | N | N | Y | Monthly |
| Spokane | WA | Spokane WA | SPK | West | Y | Y | Y | Monthly |
| Springfield | IL | Springfield IL | SPI | Midwest | N | N | Y | Monthly |
| Springfield | MO | Springfield MO | SGF | Midwest | Y | Y | Y | Monthly |
| St Augustine | FL | St Augustine FL | UST | South | N | N | Y | Monthly |
| St Cloud | MN | St Cloud MN | STC | Midwest | N | N | Y | Monthly |
| St George | UT | St George UT | STG | West | N | N | Y | Monthly |
| St Joseph | MO | St Joseph MO | STJ | Midwest | N | N | Y | Monthly |
| St Louis | MO | St Louis MO | STL | Midwest | Y | Y | Y | Weekly |
| State College | PA | State College PA | PSU | Northeast | N | N | Y | Monthly |
| Statesboro | GA | Statesboro GA | TBR | South | N | N | Y | Monthly |
| Stillwater | OK | Stillwater OK | OSU | South | N | N | Y | Monthly |
| Stockton | CA | Stockton CA | STK | West | Y | Y | Y | Monthly |
| Susanville | CA | Susanville CA | SSN | West | N | N | Y | Monthly |
| Syracuse | NY | Syracuse NY | SYR | Northeast | Y | Y | Y | Monthly |
| Tallahassee | FL | Tallahassee FL | TAL | South | Y | Y | Y | Monthly |
| Tampa | FL | Tampa FL | TPA - HIL | South | Y | Y | Y | Twice a week |
| Terre Haute | IN | Terre Haute IN | THA | Midwest | N | N | Y | Monthly |
| Texarkana | AR | Texarkana AR | TXK | South | N | N | Y | Monthly |
| Texoma | TX | Texoma TX | TXM | South | N | N | Y | Monthly |
| The Thumb | MI | The Thumb MI | THB | Midwest | N | N | Y | Monthly |
| Toledo | OH | Toledo OH | TOL | Midwest | Y | Y | Y | Monthly |
| Topeka | KS | Topeka KS | TPK | Midwest | N | N | Y | Monthly |
| Treasure Coast (Port St Lucie) | FL | Treasure Coast (Port St Lucie) FL | PSL | South | Y | Y | Y | Every two weeks |
| Tri-Cities | TN | Tri-Cities TN | TRI | South | N | N | Y | Monthly |
| Tucson | AZ | Tucson AZ | TUS | West | Y | Y | Y | Every two weeks |
| Tulsa | OK | Tulsa OK | TUL | South | Y | Y | Y | Every two weeks |
| Tuscaloosa | AL | Tuscaloosa AL | TSC | South | N | N | Y | Monthly |
| Tuscarawas | CO | Tuscarawas CO | NPH | West | N | N | Y | Monthly |
| Twin Falls | ID | Twin Falls ID | TWF | West | N | N | Y | Monthly |
| Twin Tiers | NY/PA | Twin Tiers NY/PA | TTS | Northeast | N | N | Y | Monthly |
| Tyler/East | TX | Tyler/East TX | ETX | South | N | N | Y | Monthly |
| Upper Peninsula | MI | Upper Peninsula MI | YUP | Midwest | N | N | Y | Monthly |
| Utica-Rome-Oneida | NY | Utica-Rome-Oneida NY | UTI | Northeast | N | N | Y | Monthly |
| Valdosta | GA | Valdosta GA | VLD | South | N | N | Y | Monthly |
| Ventura County | CA | Ventura County CA | OXR | West | Y | Y | Y | Monthly |
| Vermont (State) | VT | Vermont (state) | BRL | Northeast | Y | Y | Y | Monthly |
| Victoria | TX | Victoria TX | VIC | South | N | N | Y | Monthly |
| Visalia-Tulare | CA | Visalia-Tulare CA | VIS | West | N | N | Y | Monthly |
| Waco | TX | Waco TX | WCO | South | N | N | Y | Monthly |
| Washington DC | - | Washington DC | WDC - DOC | South | Y | Y | Y | Twice a week |
| Waterloo/Cedar Falls | IA | Waterloo/Cedar Falls IA | WLO | Midwest | N | N | Y | Monthly |
| Watertown | NY | Watertown NY | WTN | Northeast | N | N | Y | Monthly |
| Wausau | WI | Wausau WI | WAU | Midwest | N | N | Y | Monthly |
| Wenatchee | WA | Wenatchee WA | WEN | West | N | N | Y | Monthly |
| West Virginia (state) | WV | West Virginia (state) | WVA | South | N | N | Y | Monthly |
| Western Illinois | IL | Western IL | QCY | Midwest | N | N | Y | Monthly |
| Western Kentucky | KY | Western KY | WKY | South | N | N | Y | Twice a week |
| Western Maryland | MD | Western Maryland MD | WMD | South | N | N | Y | Monthly |
| Western Massachusetts | MA | Western Massachusetts MA | WMA | Northeast | Y | Y | Y | Monthly |
| Western Slope | CO | Western Slope CO | GJT | West | N | N | Y | Monthly |
| Wichita | KS | Wichita KS | WIC | Midwest | Y | Y | Y | Every two weeks |
| Wichita Falls | TX | Wichita Falls TX | WTF | South | N | N | Y | Monthly |
| Williamsport | PA | Williamsport PA | WPT | Northeast | N | N | Y | Monthly |
| Wilmington | NC | Wilmington NC | WNC | South | N | N | Y | Monthly |
| Winchester | VA | Winchester VA | OKV | South | N | N | Y | Monthly |
| Winston Salem | NC | Winston Salem NC | WSL | South | Y | Y | Y | Monthly |
| Worcester | MA | Worcester MA | WOR | Northeast | Y | Y | Y | Monthly |
| Wyoming (State) | WY | Wyoming (state) | WYO | West | Y | Y | Y | Monthly |
| Yakima | WA | Yakima WA | YAK | West | N | N | Y | Monthly |
| York | PA | York PA | YRK | Northeast | N | N | Y | Monthly |
| Youngstown | OH | Youngstown OH | YNG | Midwest | N | N | Y | Monthly |
| Yuba-Sutter | CA | Yuba-Sutter CA | YBS | West | N | N | Y | Monthly |
| Yuma | AZ | Yuma AZ | YUM | West | N | N | Y | Monthly |
| Zanesville/Cambridge | OH | Zanesville/Cambridge OH | ZYL | Midwest | N | N | Y | Monthly |
